# Efficient solution of Boolean satisfiability problems with digital memcomputing

**DOI:** 10.1038/s41598-020-76666-2

**Published:** 2020-11-12

**Authors:** Sean R. B. Bearden, Yan Ru Pei, Massimiliano Di Ventra

**Affiliations:** Department of Physics, University of California, San Diego, La Jolla, CA 92093 USA

**Keywords:** Engineering, Nanoscience and technology

## Abstract

Boolean satisfiability is a propositional logic problem of interest in multiple fields, e.g., physics, mathematics, and computer science. Beyond a field of research, instances of the SAT problem, as it is known, require efficient solution methods in a variety of applications. It is the decision problem of determining whether a Boolean formula has a satisfying assignment, believed to require exponentially growing time for an algorithm to solve for the worst-case instances. Yet, the efficient solution of many classes of Boolean formulae eludes even the most successful algorithms, not only for the worst-case scenarios, but also for typical-case instances. Here, we introduce a memory-assisted physical system (a digital memcomputing machine) that, when its non-linear ordinary differential equations are integrated numerically, shows evidence for polynomially-bounded scalability while solving “hard” planted-solution instances of SAT, known to require exponential time to solve in the typical case for both complete and incomplete algorithms. Furthermore, we analytically demonstrate that the physical system can efficiently solve the SAT problem in *continuous* time, without the need to introduce chaos or an exponentially growing energy. The efficiency of the simulations is related to the collective dynamical properties of the original physical system that persist in the numerical integration to robustly guide the solution search even in the presence of numerical errors. We anticipate our results to broaden research directions in physics-inspired computing paradigms ranging from theory to application, from simulation to hardware implementation.

The Boolean satisfiability problem^1^ (SAT) is an important decision problem solved by determining if a solution exists to a Boolean formula. A SAT instance is satisfiable when there exists an assignment of Boolean variables (each either TRUE or FALSE) that results in the Boolean formula returning TRUE. Apart from its academic interest, the solution of SAT instances is required in a wide range of practical applications, including, travel, logistics, software/hardware design, etc.^2^.

The SAT problem has been studied for decades, and has an important role in the history of computational complexity. Computer scientists, while categorizing the efficiency of algorithms, defined the NP class for difficult decision problems^3,4^. Some are known as *intractable* problems, meaning they are “hard” in the sense that all known algorithms cannot be bounded in polynomial time when determining if a solution exists in the worst-case scenario. The SAT problem was the first to be shown to belong to the class of NP-complete problems^3^, implying that any decision problem in NP is reducible to a SAT problem in polynomial time. There are no known polynomial time algorithms for solving an NP-complete problem, though there are exponential time algorithms that are efficient for special cases of problem structure^4^. There is a “widespread belief”^4^ that creation of a polynomial time algorithm is impossible, but this belief does not limit the realization of a polynomial *continuous-time* physical system.

NP-completeness is not exclusive to SAT, with hundreds of other NP-complete problems ranging from those of academic interest (graph theory, algebra and number theory, mathematical programming) to industry application (network design, data storage and retrieval, program optimization)^4^. If a polynomial time algorithm can solve any NP-complete problem class, then all NP problems can be computed efficiently. The 3-SAT problem is NP-complete and a special case of SAT^4^. Randomly-generated 3-SAT instances are known to be difficult to many solution methods because they lack an exploitable problem structure. For instance, one lauded algorithm, survey inspired decimation (SID), performs well on large instances of uniform random 3-SAT in the “hard regime”^5^, but performs poorly in what is known as the “easy regime”^6^. We focus on the 3-SAT problem in the following due to it being a subclass of SAT with a consistent formulaic representation (three literals per clause).

## Physics-inspired approach to computing

A research direction that has been far less explored concerns the solution of SAT using non-quantum *dynamical systems*^7–10^. The idea behind this approach is that the solutions of the SAT instance are mapped into the equilibrium points of a dynamical system. If the initial conditions of the dynamics belong to the basin of attraction of the equilibrium points, then the dynamical system will have to “fall” into these points. The approach is *fundamentally* different from the standard algorithms because dynamical systems perform computation in *continuous time*. Numerical simulation of continuous-time physical systems, an algorithm, requires the discretization of time to integrate the ordinary differential equations (ODEs) representing the physical system. As such, the dynamical-systems approach is ideally suited for a hardware implementation.

The authors of Ref.^8^ have shown that an appropriately designed dynamical system can find the solutions of hard 3-SAT instances in continuous polynomial time, however, at a cost of *exponential* energy fluctuations. The reason for this exponential energy cost can be traced to the transient *chaotic* dynamics of the dynamical systems proposed in Ref.^8^. As the problem size grows, the chaotic behavior translates into an exponentially increasing number of integration steps required to find the equilibrium points of the corresponding ODEs.

**Figure 1:**
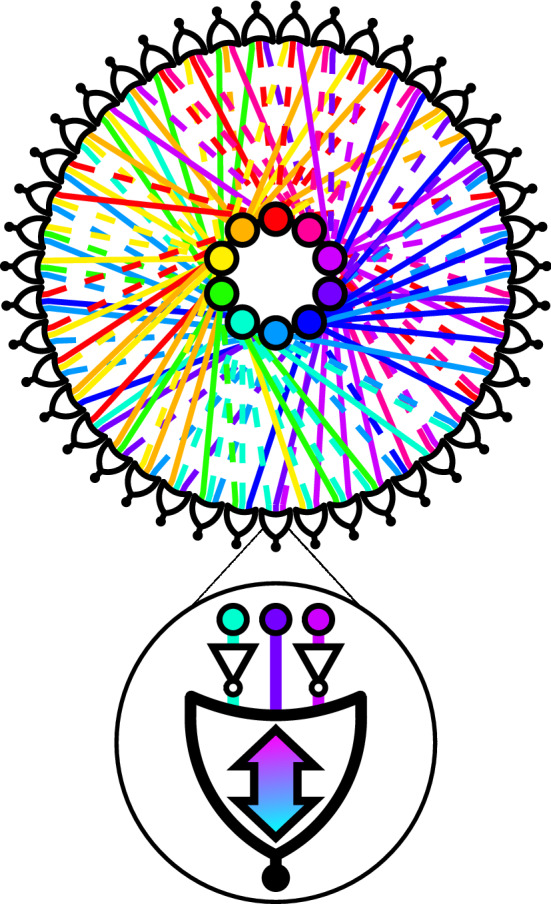
Schematic of a self-organizing logic circuit representing a 3-SAT instance. The circuit is created from the constraints of a 3-SAT formula consisting of $N=10$ variables, and $M=43$ clauses. The formula is converted into 10 voltage nodes (inner nodes) and 43 self-organizing OR gates^11^. The black nodes (outer nodes) traditionally associated with the output of the OR gates are fixed to TRUE to enforce the constraints. Dashed lines in the circuit represent NOT gates on the OR gate terminals. Ignoring the black nodes, the circuit can be interpreted as a factor graph with the gates becoming function nodes (see also Fig. 3). The clause represented by the highlighted self-organizing OR gate is $({\bar{y}}_i \vee y_j \vee {\bar{y}}_k)$, where NOT gates invert the polarity of the voltages. The double-headed arrow indicates this is a self-organzing logic gate with no distinction between an input and an output (terminal agnosticism). The circular representation of the linear circuit is a reminder that the ordering of gates is irrelevant to the solution search.

## The digital memcomputing approach

In recent years, a different physics-inspired computational paradigm has been introduced, known as *digital memcomputing*^10,12^. Digital memcomputing machines (DMMs) are non-linear dynamical systems specifically designed to solve constraint satisfaction problems, *e.g.*, 3-SAT, with the assistance of memory^10^ (Fig. 1). The only equilibrium point(s) of the DMM is the solution(s) of the original problem. However, unlike previous work, DMMs are designed so that they have no other equilibrium points; see Sect. VI.D of the supplementary material (SM). Additionally, the dynamics will never enter a periodic orbit or a state of chaos^13^ (see Sect. IX of SM).

The ability of continuous time dynamics to perform the solution search without resorting to chaotic dynamics results in efficient *simulations* (an algorithmic implementation) of DMMs using computationally-inexpensive integration schemes and modern computers. In addition, it was shown that DMMs find the solution of a given problem by employing topological objects, known as instantons, that connect critical points of increasing stability in the phase space^14,15^ (see Sect. XI of SM). Simulations found the DMMs then self-tune into a critical (*collective*) state which persists for the whole transient dynamics until a solution is found^16^. It is this critical branching behavior that allows DMMs to explore *collective* updates of variables during the solution search, without the need to check an exponentially-growing number of states. This is in contrast to local-search algorithms which are characterized by a “small” (not collective) number of variable updates at each step of the computation^17^.

Here, we introduce a physical DMM to find solutions of the 3-SAT problem. (So as to facilitate the reading of our paper, we have contained the mathematical description of our physical DMM in the section below.) We then perform numerical simulations of the ODEs (discretized time) of the DMM to solve random 3-SAT instances with planted solutions. These instances are generated with a clause distribution control (CDC) procedure, known to require exponentially growing time to solve in the typical case for both complete and incomplete algorithms^18^. The CDC instances have found use as benchmarks in recent years of SAT competitions (satcompetition.org)^19–21^. The simulations have been performed using a forward-Euler integration scheme^22^ with an adaptive time step, implemented in MATLAB R2019b with each solution attempt run on a single logic core of an AMD EPYC 7401 server (see also Sect. II of SM).

**Figure 2:**
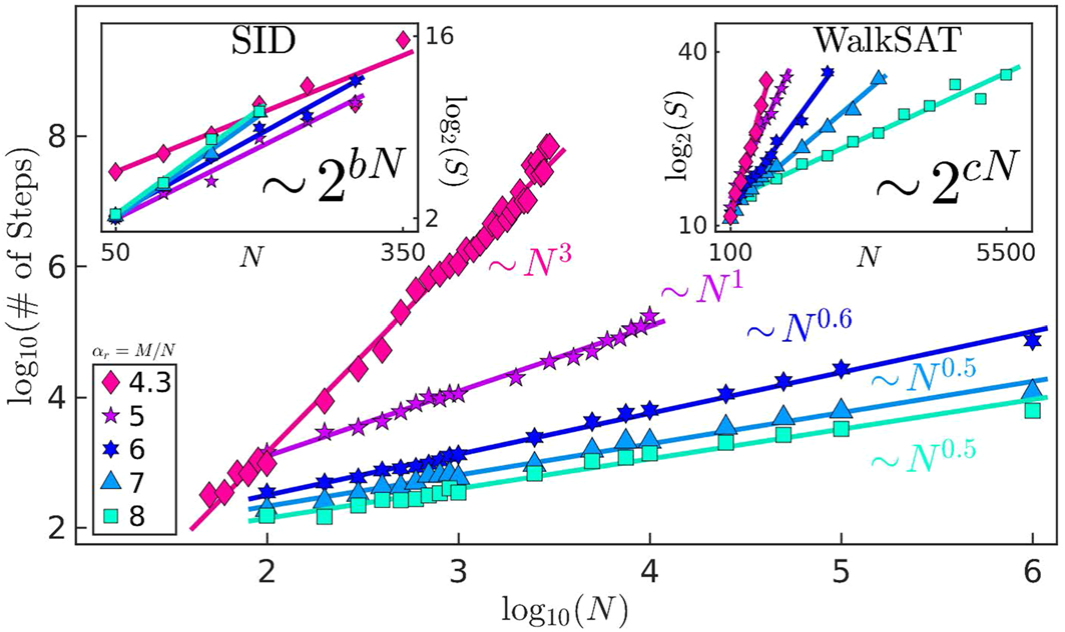
Typical case scalability of 3-SAT instances at fixed clause-to-variable ratio. In the main panel, we use our DMM algorithm to attempt to solve 100 planted-solution instances of 3-SAT per pair of $\alpha _r$ (clause-to-variable ratio) and *N* (number of variables). When we achieve more than 50 instances solved, we find power-law scalability of the median number of integration steps (typical case) as the number of variables, *N*, grows. (In the SM, we show many data points are comprised of 90 or more instances solved within the allotted time.) The exponent values ($\sim \!N^a$) are $a_{4.3}=3.0\pm 0.1$, $a_{5}=1.00\pm 0.05$, $a_{6}=0.63\pm 0.03$, $a_{7}=0.48\pm 0.03$, and $a_{8}=0.46\pm 0.04$. The insets show exponential scalability for a stochastic local-search algorithm (WalkSAT) and a survey-inspired decimation procedure (SID) on the same instances. (S is for number of steps.) Notice the scalability for SID has a trend opposite that seen in the DMM and WalkSAT. This is expected when one considers the increase in factor graph loops as $\alpha _r$ grows. For the SID scaling of $\alpha _r=4.3$, the $N=350$ did not achieve a median number of solutions, and is thus a lower bound. Parameters of the scaling for SID: $b_{4.3}=(3\pm 1)\times 10^{-2}$, $b_{5}=(3.7\pm 0.7)\times 10^{-2}$, $b_{6}=(4.1\pm 0.6)\times 10^{-2}$, $b_{7}=(5\pm 1)\times 10^{-2}$, and $b_{8}=(5\pm 1)\times 10^{-2}$; for WalkSAT: $c_{4.3}=(3.2\pm 0.3)\times 10^{-2}$, $c_{5}=(1.9\pm 0.2)\times 10^{-2}$, $c_{6}=(1.2\pm 0.1)\times 10^{-2}$, $c_{7}=(7.5\pm 0.6)\times 10^{-3}$, and $c_{8}=(4.1\pm 0.5)\times 10^{-3}$.

We compare our results with those obtained from two well-known algorithms: WalkSAT, a stochastic local-search procedure^23^, and survey-inspired decimation, a message-passing procedure utilizing the cavity method from statistical physics^5^. (in Sect. II of the SM we also compare with the winner of a recent SAT competition and AnalogSAT^24^). Comparison is achieved via scalability of some indicator vs. the problem size. As expected, both algorithms show an exponential scaling for the CDC instances (Fig. 2). Our simulations instead show a power-law scalability of integration steps ($\sim \!N^{a}$) for typical cases, where the typical case is inferred from the median number of integration steps.

Finally, we show that the dynamics is capable of finding satisfying variable assignments for 3-SAT in polynomially-bounded (linear or sub-linear) *continuous* time without the need of an exponentially increasing energy cost demonstrated via certain dissipative and topological properties of the system (see Secs. X-XI of SM).

While the reported numerical and analytical results do not resolve the famous P vs. NP debate, (which, incidentally, is formulated for Turing machines, that compute in *discrete*, not continuous time) they show the tremendous advantage of physics-based approaches to computation over traditional algorithmic approaches.

## DMM for 3-SAT

The 3-SAT formula is constructed by applying conjunction (AND), disjunction (OR), and negation (NOT) operations to Boolean variables (TRUE or FALSE), with parentheses used to indicate the order of operations^25^. A formula contains *N* Boolean variables ($y_i$), *M* clauses, and 3*M* literals. Each clause (constraint) consists of three literals connected by logical OR operations, i.e., $(l_i\vee l_j\vee l_k)$, where a literal, $l_i$, is simply one of the Boolean variables ($l_i=y_i$) or its negation ($l_i=\bar{y_i}$). A clause is satisfied if at least one literal is TRUE (OR operations), and the formula is satisfiable if all clauses (AND operations) are simultaneously satisfied. The complexity of the problem emerges from the interaction among constraints, and is observed in the well-studied easy-hard-easy transition in 3-SAT, where easy and hard regimes are identified by the ratio $\alpha _r=M/N$, with the complexity peak (hardest instances) occurring around $\alpha _r=4.27$^26^.

To construct a DMM that finds a satisfying assignment for 3-SAT we follow the general procedure outlined in Ref.^12^. To begin, the Boolean variables, $y_i$, are transformed into continuous variables for use in the DMM. The continuous variables can be realized in practice as voltages on the terminals of a self-organizing OR gate^10^. Such a gate can influence its terminals to push voltages towards a configuration satisfying its OR logic *regardless* of whether the signal received by the gate originates from the traditional input or the traditional output (see Fig. 1). The voltages are bounded, $v_i\in [-1,1]$, with Boolean values recovered by thresholding: TRUE if $v_i>0$, FALSE if $v_i<0$, and ambiguous if $v_i=0$. To perform the logical negation operation on the continuous variable, one trivially multiplies that quantity by $-1$. The self-organizing logic circuit that comprises the DMM is built by connecting all of the self-organizing OR gates (see Fig. 1). See Sect. III.A of SM for an extended discussion of the thresholding procedure for the voltages.

Next, we interpret a Boolean clause as a dynamical constraint function, with its state of satisfaction determined by the voltages. The *m*-*th* Boolean clause, $(l_{i,m} \vee l_{j,m} \vee l_{k,m})$, becomes a constraint function,

1 $$\begin{aligned} C_{m}(v_{i},v_{j},v_{k}) = \frac{1}{2}\min [(1- q_{i,m}v_i),(1 - q_{j,m}v_j),(1 - q_{k,m}v_k)], \end{aligned}$$

where $q_{i,m}=1$ if $l_{i,m}=y_i$, and $q_{i,m}=-1$ if $l_{i,m}={\bar{y}}_i$. The function is bounded, $C_m \in [0,1]$, and a clause is necessarily satisfied when $C_m<1/2$. The instance is solved when $C_m<1/2$ for all clauses. By thresholding the constraint function we avoid the ambiguity associated with $v_i=0$. If some voltage is ambiguous ($v_j=0$) and all clauses are satisfied, then any Boolean assignment to $y_j$ will be valid in that configuration. The use of a minimum function in $C_{m}$ preserves an important property of 3-SAT. A clause is a constraint, and, by itself, a clause can only constrain one variable (via its literal). (Note that the minimum operation introduces some form of discontinuity to the dynamical system, for which we develop the formalism to study in Secs. IV and V of SM.) The values of two literals are irrelevant to the state of the clause if the third literal results in a satisfied clause.

Finally, a DMM employs memory variables to assist with the computation^10,12^. The memory variables transform equilibrium points that do not correspond to solutions of the Boolean formula into unstable points in the voltage space (see Sect. VIII of SM), leaving the solutions of the 3-SAT problem as the only minima. We choose to introduce two memory variables per clause: short-term memory, $x_{s,m}$, and long-term memory, $x_{l,m}$. The terminology intuitively describes the behavior of their dynamics. For the short-term memory, $x_{s,m}$ lags $C_{m}$, acting as an indicator of the recent history of the clause. For the long-term memory, $x_{l,m}$ collects information so it can “remember” the most frustrated clauses, weighting their dynamics more than clauses that are “historically” easily satisfied. Both the number and type of memory variables, as well as the form of the resulting dynamical equations, are not unique provided neither chaotic dynamics nor periodic orbits are introduced^12^.

We choose for the dynamics of voltages and memory variables the following,

2 $$\begin{aligned}&{\dot{v}}_n = \sum _{m} x_{l,m}x_{s,m}G_{n,m}(v_{n},v_{j},v_{k}) + (1+\zeta x_{l,m})(1-x_{s,m})R_{n,m}(v_{n},v_{j},v_{k}), \end{aligned}$$

3 $$\begin{aligned}&{\dot{x}}_{s,m} = \beta (x_{s,m}+\epsilon )(C_m(v_{i},v_{j},v_{k})-\gamma ), \end{aligned}$$

4 $$\begin{aligned}&{\dot{x}}_{l,m} = \alpha (C_m(v_{i},v_{j},v_{k})-\delta ), \end{aligned}$$

5 $$\begin{aligned}&G_{n, m}(v_{n},v_{j},v_{k}) = \frac{1}{2} q_{n,m} \min [(1 -q_{j,m}v_j),(1 - q_{k,m}v_k)], \end{aligned}$$

6 $$\begin{aligned}&R_{n,m}(v_{n},v_{j},v_{k}) = {\left\{ \begin{array}{ll} \frac{1}{2}( q_{n,m}-v_n), & C_m(v_{n},v_{j},v_{k})=\frac{1}{2}(1- q_{n,m}v_n), \\ 0, & \text {otherwise}, \end{array}\right. } \end{aligned}$$

where $G_{n,m}$ and $R_{n,m}$ equal 0 when variable *n* does not appear in clause *m*, and the summation is taken over all constraints in which the voltage appears. The memory variables are *bounded*, with $x_{s,m}\in [0,1]$ and $x_{l,m}\in [1,10^4M]$. The boundedness of voltage and memory variables implies that there are no diverging terms in the above equations (see Sect. VI.B of SM).

The parameters $\alpha$ and $\beta$ are the rates of growth for the long-term and short-term memory variables, respectively. Each memory variable has a threshold parameter used for evaluating the state of $C_m$, and the two parameters are restricted to obey $\delta<\gamma <1/2$. (This also guarantees that there is a sufficiently large basin of attraction for the solutions. See Sect. VII of SM for a detailed explanation.). Equation (3) has a small, strictly-positive parameter, $0<\epsilon \ll 1$, to remove the spurious solution ($x_{s,m}=0$). However, $\epsilon$ additionally serves as a trapping rate in the sense that smaller values of $\epsilon$ make it more difficult for the system to flip voltages when some $C_m$ begins to grow larger than $\gamma$.

In Eq. (2), the first term in the summation is a “gradient-like” term, the second term is a “rigidity” term^16^. The gradient-like term attempts to influence the voltage in a clause based on the value of the other two voltages in the associated clause. Consider the two extremes: if the minimum results is $G_{i,m}=1$, then $v_i$ needs to be influenced to satisfy the clause. Conversely, if the minimum gives $G_{i,m}=0$, then $v_i$ does not need to influence the clause state (see Sect. II.A of SM).

The purpose of the three rigidity terms for one constraint is to attempt to hold one voltage at a value satisfying the associated *m*-*th* clause, while doing nothing to influence the evolution of the other two voltages in the constraint. Again, this aligns with the 3-SAT interpretation that a clause can only constrain one variable. The short-term memory variable acts as a switch between gradient-like dynamics and rigid dynamics. During the solution search, $G_m$ will seek to influence three voltages until clause *m* has been satisfied. Then, as $x_{s,m}$ decays to zero, $R_m$ takes over. The long-term memory variables weight the gradient-like dynamics, giving greater influence to clauses that have been more frustrated during the solution search. The rigidity is also weighted by $x_{l,m}$, but reduced by $\zeta$.

**Figure 3:**
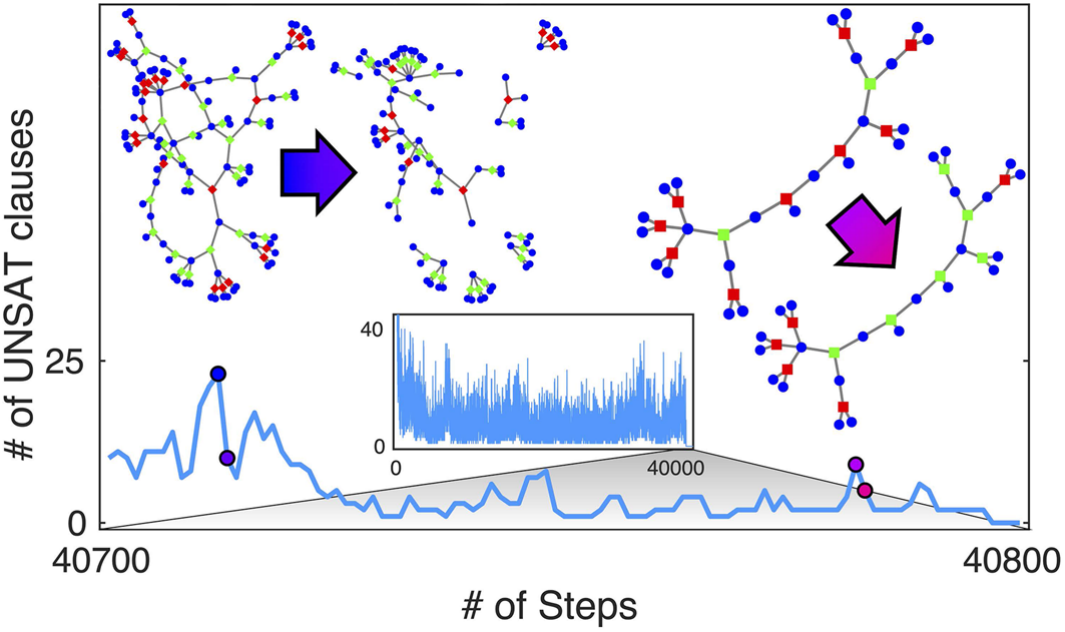
Time evolution of a typical DMM simulation showing collective updates to the solution search. The figure highlights one solution attempt of a CDC instance of size $N=500$ at $\alpha _r=4.3$. The inset shows the number of unsatisfied clauses during the entire solution search. The main panel zooms in on the search as the solution is approached. We choose two single integration step transitions and explore the local factor graph. The circles are the variable nodes (blue), and squares are function nodes (red if unsatisfied, green if recently unsatisfied). The transition at left is characterized by 13 clauses becoming satisfied, the transition at right results in 4 clauses becoming satisfied. Neither transition results in satisfied clauses becoming unsatisfied.

## Numerical results and discussion

It is important to realize that any simulation of a dynamical system is an algorithm because the continuous-time dynamics of the system must be discretized. Identifying our simulation as an algorithm invites a method to compare our results with those of popular algorithms, specifically, WalkSAT^23^ and survey inspired decimation^5^. Before we compare results, we need a general definition of a step.

We define an algorithmic step to be all the computation that occurs between checks of satisfiability. The WalkSAT algorithm flips one variable at a time then checks the satisfiability of the formula. Therefore, a WalkSAT step is a single variable flip. SID uses WalkSAT as part of its solution search, so the interpretation of steps is the same when SID uses WalkSAT. Prior to entering into WalkSAT, SID performs a message-passing procedure known as survey propagation^5^. In the SID implementation we used^27^ there is no check for satisfiability during the decimation procedure, so we generously identify the entire survey propagation with decimation as a single step. Our DMM algorithm checks the satisfiability of the formula after each time step of the integration. Of course, the amount of computation within a step may vary greatly based on the algorithm, but this does not affect comparison of the scalability. In fact, if an algorithm is exponential in the number of steps, then the amount of computation within a step cannot improve its scalability. For our DMM, each step has a constant amount of computation per time step of integration. With this definition of an algorithmic step, we have a method to meaningfully compare the different algorithms.

We can now test these approaches on CDC instances with planted solutions. In Sect. III.C of the SM, we give an account of how these instances are generated, and why they are difficult to solve. Here, we just note that difficult CDC instances are created when $\alpha _r>4.25$ and $0.077<p_0<0.25$, where $p_0$ is the probability that the planted solution results in a clause with zero false literals^18^. We have performed no preprocessing on the 3-SAT instances to reduce their size, not even the removal of pure literals (those appearing wholly negated or unnegated)^28^.

We numerically integrated Eqs. (2), (3), and (4) with the forward-Euler method using an adaptive time step, $\Delta t\in [2^{-7},10^3]$. For parameters, we have used $\alpha = 5$, $\beta = 20$, $\gamma =1/4$, $\delta = 1/20$, and $\epsilon = 10^{-3}$. For high ratio, $\alpha _r\ge 6$, we find $\zeta =10^{-1}$ to provide better scaling results. For ratios that approach the complexity peak, we used $\zeta =10^{-2}$ for $\alpha _r=5$, and $\zeta =10^{-3}$ for $\alpha _r=4.3$. In Fig. 2, we report the results for CDC instances generated with $p_0=0.08$. In our simulations, we expectedly find the difficulty of CDC instances increases with increasing $p_0$ (see Sect. II in SM).

In Fig. 2, for the problem sizes tested, we find a power-law scaling for the median number of integration steps for the simulations of DMMs. We also find that integration time variable (*t*), CPU time, and long-term memory ($x_l$) are bounded by a polynomial scaling, and the average step size shows power-law decay (see Sect. II.C of SM). The optimized WalkSAT algorithm^29^ we have used instead exhibits an exponential scaling at relatively small problem sizes, confirming the previous results of Ref.^18^. An exponential scaling is also observed for the SID algorithm^27^.

The CDC instances are formulated to confuse stochastic local-search algorithms, so the exponential scaling of WalkSAT is expected (right inset Fig. 2). To understand the exponential performance of SID (left inset Fig. 2), we need to understand the success of SID on random 3-SAT. When generating uniform random 3-SAT at the complexity peak with a general method (no planted solutions), the typical case can be exploited by SID due to the existence of treelike structures in the factor graph^30^. (For those unfamiliar with factor graphs, if the factor graph was a tree, then one would be able to visually, thus easily, find the solution from the graph^31^.) However, as demonstrated in Fig. 2, SID performs poorly when given a 3-SAT instance with a factor graph that is not locally treelike. It is also known that SID performs poorly at high ratios ($\alpha _r \gtrsim 4.25$)^6^, as loops in the factor graph become more common, explaining the opposite scaling trend seen in Fig. 2.

To further confirm that the usefulness of our DMM algorithm on CDC instances is independent of our generation of formulae, we have solved generalized CDC instances^19^ used in the 2017^20^ and 2018^21^ SAT competitions (satcompetition.org). Our modified competition DMM solves *all* tested competition CDC instances on its first attempt with random initial conditions, and does so within the 5000-second timeout established by the competition (see Sect. II.E of SM). We find the overhead of numerical simulations of ODEs does not forbid our DMM from being competitive due to the use of the forward-Euler integration scheme.

### Long-range order and analytical properties of DMMs for 3-SAT

We finally show that collective behavior (*long-range order*)^14,15^ in DMMs is responsible for the observed efficiency in the solution search. In order to do this, it is helpful to visualize subgraphs of the factor graph generated from a 3-SAT instance. In Fig. 3, we visualize the change in state of local factor graphs during a single time step of integration as our DMM approaches a solution. It is apparent that the system explores many paths in the factor graph, collecting information as it does. However, unlike SID, when the DMM explores a path leading to contradiction it can *correct itself*. The factor graphs shown in Fig. 3 only include clauses (function nodes) that are unsatisfied (red) or recently unsatisfied (green), and all variable nodes connected to these clauses. A clause, *m*, is identified as recently unsatisfied if the short-term memory is $x_{s,m}>0$ but the clause is currently satisfied. The factor graph transitions show that *collective events* occur that satisfy multiple clauses. This is in agreement with many results on DMMs for different types of problems^14,32^. Additionally, the factor graph transition on the left of Fig. 3 breaks up the graph into smaller, disconnected factor graphs, making the search exponentially more efficient.

As anticipated, to strengthen these numerical results, we have also analytically demonstrated that the dynamics described by Eqs. (2), (3), and (4) terminate *only* when the system has found the solution to the 3-SAT problem (namely the phase space has only saddle points and the minima corresponding to the solution of the given problem; Secs. VI and VII of SM). In addition, neither periodic orbits nor chaos can coexist if solutions of the 3-SAT are present (Sect. IX of SM). Finally, using supersymmetric topological field theory, we have demonstrated that the continuous-time dynamics (physical implementation) reach the solution of a 3-SAT instance, for a fixed $\alpha _r$, in linear or sub-linear *continuous* time, irrespective of the difficulty of the instance (Sect. XI of SM).

However, note that such a scalability *does not* necessarily translate to the same scalability of the *numerical* integration of Eqs. (2), (3), and (4), where the discretization of time is necessary. Nevertheless, due to the absence of chaos, we empirically find that the scalability of our numerical simulations is still polynomially bounded for typical-case CDC instances.

## Conclusions

We have presented an efficient dynamical-system approach to solving Boolean satisfiability problems. Along with arguments for polynomial-time scalability in *continuous* time, we have found that the *numerical* integration of the corresponding ODEs show power-law scalability for typical cases of 3-SAT instances which required exponential time to solve with successful algorithms. The efficiency derives from *collective* updates to the variables during the solution search (*long-range order*).

In contrast to previous work^8^, our dynamical systems do not suffer from exponential fluctuations in the energy function due to chaotic behavior. The dynamical systems we propose find the solution of a given problem without ever entering a chaotic regime, by virtue of the variables being bounded. The implication is that a hardware implementation of DMMs would only require a polynomially-growing energy cost. Our work then also serves as a counterexample to the claim of Ref.^8^ that chaotic behavior is necessary for the solution search of hard optimization problems. In fact, we find chaos to be an undesirable feature for a scalable approach (See Sect. II.F of SM).

Although these analytical and numerical results do not settle the famous P vs. NP question, they show that appropriately designed physical systems are very useful tools for new avenues of research in constraint satisfaction problems.

## Supplementary information

Supplementary information.

## Data Availability

All instances used to generate all figures in this paper are available upon request from the authors.
